# Brain metabolic subtypes in Long Covid individuals from a Brazilian cohort

**DOI:** 10.1002/alz.093278

**Published:** 2025-01-09

**Authors:** Arthur Viana Jotz, Marco De Bastiani, Luiza Santos Machado, Thomas Hugentobler Schlickmann, Maiele Dornelles Silveira, Joana Emilia Senger, Wyllians Vendramini Borelli, João Pedro Ferrari‐Souza, Guilherme Povala, Ana Paula Bornes da Silva, João Pedro Uglione da Ros, Guilherme Bastos de Mello, Matheus Fakhri Kadan, Matheus Fakhri Kadan, Graciane Radaelli, Daniele de Paula de Paula Faria, Artur Martins Coutinho, Mychael V Lourenco, Micaela A Hernández, Ismael Luis Calandri, Tharick A. Pascoal, Cristina Sebastião Matushita, Ricardo Benardi Soder, Artur Francisco Schumacher‐Schuh, Diogo O. Souza, Jaderson Costa da Costa, Débora Guerini de Souza, Eduardo R. Zimmer

**Affiliations:** ^1^ Federal University of Rio Grande do Sul, Porto Alegre, RS Brazil; ^2^ UFRGS, Porto Alegre, PA Brazil; ^3^ Universidade Federal de Pelotas, Pelotas Brazil; ^4^ Universidade Federal do Rio Grande do Sul, Porto Alegre, Rio Grande do Sul Brazil; ^5^ Federal University of Rio Grande do Sul, Porto Alegre, Rio Grande do Sul Brazil; ^6^ UFRGS, Porto Alegre Brazil; ^7^ Federal University of Rio Grande do Sul, Brazil, Porto Alegre, Rio Grande do Sul Brazil; ^8^ Memory Center, Hospital Moinhos de Vento, Porto Alegre, RS Brazil; ^9^ Clinical Hospital of Porto Alegre, Porto Alegre, Rio Grande do Sul Brazil; ^10^ Brain Institute of Rio Grande do Sul (InsCer), PUCRS, Porto Alegre, Rio Grande do Sul Brazil; ^11^ University of Pittsburgh, Pittsburgh, PA USA; ^12^ Federal University of Rio Grande do Sul, Brazil, Porto Alegre, RS Brazil; ^13^ Lutheran University of Brazil, Canoas, Rio Grande do Sul Brazil; ^14^ Pontifícia Universidade Católica do Rio Grande do Sul, Porto Alegre, Rio Grande do Sul Brazil; ^15^ Brain Institute, RS, Porto Alegre, Rio Grande do Sul Brazil; ^16^ USP, São Paulo Brazil; ^17^ University of São Paulo Medical School, São Paulo, São Paulo Brazil; ^18^ Center of Nuclear Medicine, Institute of Radiology, Hospital das Clínicas, Faculdade de Medicina da Universidade de São Paulo, São Paulo, São Paulo Brazil; ^19^ Universidade Federal do Rio de Janeiro, Rio de Janeiro Brazil; ^20^ Federal University of Rio de Janeiro, Rio de Janeiro, Rio de Janeiro Brazil; ^21^ Institute of Medical Biochemistry Leopoldo de Meis, Federal University of Rio de Janeiro, Rio De Janeiro, Rio de Janeiro Brazil; ^22^ Fleni, Buenos Aires, Buenos Aires Argentina; ^23^ Alzheimer Center Amsterdam, Amsterdam UMC, Amsterdam Netherlands; ^24^ Amsterdam Neuroscience, Neurodegeneration, Amsterdam Netherlands; ^25^ Fleni, Buenos Aires Argentina; ^26^ Amsterdam Neuroscience, Brain Imaging, Amsterdam Netherlands; ^27^ Brain Intitute, RS, Porto Alegre, Rio Grande do Sul Brazil; ^28^ Universidade Federal do Rio Grande do Sul, Porto Alegre Brazil; ^29^ Brain Institute of Rio Grande do Sul ‐ Pontifícia Universidade Católica do Rio Grande do Sul, Porto Alegre, Rio Grande do Sul Brazil; ^30^ Brain Institute of Rio Grande Do Sul, PUCRS, Porto Alegre, RS Brazil; ^31^ McGill Centre for Studies in Aging, Montreal, QC Canada

## Abstract

**Background:**

The Coronavirus disease 2019 (COVID‐19), caused by SARS‐CoV‐2, is one of the biggest health concerns of the century. Long COVID is one of the major sequelae from the infection and include persistent neurological manifestations. Brain images study suggest that Long COVID patients present distinct brain metabolic alterations. However, whether brain subtypes of Long COVID exist remains unclear. Here, we aimed to evaluate metabolic patterns in FDG‐PET imaging in a Brazilian cohort of individuals presenting with Long COVID.

**Method:**

A total of 37 adult Brazilian individuals presenting with Long COVID symptoms were scanned with FDG positron emission tomography (FDG‐PET) and its standardized uptake value ratio (SUVr normalized by the whole brain) was obtained. Then, 89 volumes of interest (VOIs) were extracted and used as input features for a principal component analysis (PCA). Average silhouette method was used to determine the optimal number of clusters. K‐means clustering was used to split the dataset into a set of K groups. We employed ANCOVA and Tukey tests to compare the groups. Statistical analysis was made in the R environment, significance set at p <0.05.

**Result:**

PCA analysis identified three different FDG‐PET subtypes: C1 (n=4), C2 (n=6), and C3 (n=27). In the ANCOVA comparison, the regions with the highest increase in the mean regional SUVr were left lingual gyrus for C1 compared to C2 and C3, and corpus callosum for C2 against C3. Conversely, the greatest regional decrease observed were: left caudate nucleus for C1 compared to C2 and right globus pallidus for C1 and C2 compared to C3.

**Conclusion:**

These preliminary findings suggest that Long COVID individuals present a predominant metabolic signature (C3) but at least two variants. Our results corroborate that Long COVID is a heterogeneous condition that affects individuals in a different manner. More studies are needed to understand the regional brain vulnerability to Long COVID.

